# Therapeutic Potential of *Brassica carinata* Microgreens Extract in Alleviating Symptoms of Type 2 Diabetes in Wistar Rats

**DOI:** 10.1002/fsn3.4635

**Published:** 2025-01-06

**Authors:** Lilian Nakakaawa, Ifeoluwa D. Gbala, Joel L. Bargul, Xavier Cheseto, John M. Wesonga

**Affiliations:** ^1^ Department of Molecular Biology and Biotechnology Pan African University Institute for Basic Sciences Technology, and Innovation (PAUSTI) Nairobi Kenya; ^2^ Department of Life and Physical Sciences Bugema University Kampala Uganda; ^3^ International Centre for Genetic Engineering and Biotechnology Trieste Italy; ^4^ International Centre of Insect Physiology and Ecology (Icipe) Nairobi Kenya; ^5^ Department of Biochemistry Jomo Kenyatta University of Agriculture and Technology (JKUAT) Nairobi Kenya; ^6^ Department of Horticulture and Food Security JKUAT Nairobi Kenya

**Keywords:** blood glucose levels, *Brassica carinata* microgreens, insulin resistance, lipid metabolism, type 2 diabetes mellitus

## Abstract

Microgreens of Brassica plants have attracted increasing research interest in the management of the prevailing epidemic of Type 2 diabetes mellitus (T2DM) because of their high nutritional value. This study evaluated the antidiabetic effects of *Brassica carinata* Microgreens Ethanolic Extract (BMEE) in type‐2 diabetic rats. For the normoglycemic assay, rats were divided into five groups and received a single oral dose of 100, 250, and 500 mg/kg of BMEE while the control groups received distilled water and Glibenclamide. Fasting blood glucose (FBG) levels were determined on a weekly basis for 28 days in diabetic rats after treatment with BMEE at 250 and 500 mg/kg dosage levels. Oral glucose tolerance test (OGTT), serum insulin levels, lipid profile and messenger RNA expression levels of Insulin receptor substrate 1 (*IRS‐1*), Glucose transporter 2 (*GLUT2*), and Nuclear factor kappa light‐chain enhancer of activated B cells (*NFKβ*) genes were determined. BMEE did not induce hypoglycemic effects in rats with normal blood glucose levels, but induced antidiabetic activities in the experimental type‐2 diabetic rats. BMEE lowered FBG levels, increased oral glucose tolerance, increased insulin sensitization, and reduced insulin resistance. Treatment of diabetic rats with BMEE increased lipid metabolism and relatively higher expression levels of *IRS‐1* and *GLUT2* genes, and led to reduced expression levels of *NFKβ* in the liver. Overall, this study reports that BMEE has potential as a nutraceutical to be utilized in the management of T2DM.

## Graphical abstract

*Brassica carinata* microgreens exhibited antidiabetic activities in hyperglycemic conditions of a type 2 diabetes model. Treatment lowered fasting blood glucose levels, increased oral glucose tolerance, reduced insulin resistance, and increased lipid metabolism. Treatment upregulated gene expression of IRS‐1, GLUT2 while downregulating gene expression of NFKβ in the liver.

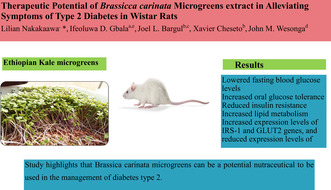

## 1 Introduction

Type 2 diabetes mellitus (T2DM) is a diverse set of chronic disorders involving the metabolism of carbohydrates, lipids, and proteins marked by hyperglycemia, insulin resistance, and impaired glucose tolerance (Zhou et al. 2023). The main clinical manifestation of T2DM, hyperglycemia, is linked to the development of diabetic complications such as vision impairment, cardiac arrest, stroke, renal dysfunction, and lower limb amputation (Karuranga et al. 2022). Diabetes mellitus affects both glucose and lipid metabolism, mainly by causing insulin resistance and influencing related hormones. This disruption results in symptoms like high blood glucose (hyperglycemia) and elevated lipid levels (hyperlipidemia). The persistent metabolic imbalances, along with possible inflammatory and hormonal shifts, can exacerbate mood disorders, leading to a higher prevalence of depressive symptoms among individuals with diabetes (Karuranga et al. 2022). This disease continues to pose a significant public health challenge, particularly in countries with low to moderate incomes, owing to the increasing premature mortality rates associated with it (Lin et al. 2020). According to the International Diabetes Federation Atlas report, in the year 2021, it was estimated that more than 537 million individuals were affected by diabetes with projections showing that this prevalence could reach 783.2 million by 2045 (Ofosu et al. 2023). Annually, there is an estimation of over 5 million deaths due to diabetes where patients suffer from intense physical and mental distress leading to significant economic and social burdens (Hu et al. 2023). Notwithstanding the notable progress made in the management of T2DM, the disease remains the second leading cause of health‐related loss of longevity worldwide (Andreadi et al. 2023).

Current interventions for T2DM include (a) pharmacological measures such as insulin prescriptions, oral antidiabetic drugs, and (b) non‐pharmacological approaches, which include diet, exercise, and lifestyle management. However, the key challenge to these pharmacological interventions is the high cost of self‐management, severe side effects, decreasing efficacy, inaccessibility, and life‐long therapy of the antidiabetic drug (Kalsi et al. 2015; Obakiro et al. 2023). The major drawbacks in the current antidiabetic interventions have fueled efforts in search of effective and safe alternative therapeutic interventions for T2DM. As such, trends are pointing toward the use of biotherapeutics such as functional foods for the management of T2DM.

*Brassica carinata* A. Braun commonly referred to as Ethiopian kale is a widely cultivated and consumed African Indigenous Vegetable in eastern and southern Africa (Hagos et al. 2020). Ethiopian kale is part of the *Brassicaceae* family, which encompasses certain plant species that have received significant attention as microgreens and functional foods due to their abundance of phytochemicals which offer both nutritional and medicinal benefits (El‐Nakhel et al. 2021; Martínez‐Ispizua et al. 2022).

Microgreens are an emerging class of functional foods that are receiving scientific attention particularly due to their high nutraceutical potential (Partap et al. 2023). Microgreens are young immature plants that are between the sprout and baby greens stages of growth generated from the seeds of vegetables, herbs or cereals with the appearance of leaves between 7 and 21 days after planting (Jambor et al. 2022; Wojdyło et al. 2020). Extensive studies on microgreens as potential candidates in the management of diet‐controlled diseases like diabetes have been conducted, and more studies are still ongoing. Microgreens of *Brassica* plants and other plants have been reported to have antidiabetic activities and could be utilized in the management of T2DM. Microgreens of *Brassica oleracea* L. var. capitata were reported to improve the lipid metabolism of mice fed on food rich in fats (Huang et al. 2022). *Hordeum vulgare* microgreens were shown to reduce reproductive disorders, oxidative stress, and the effect of aflatoxin in diabetic rats (Mohamed et al. 2022). *Brassica oleracea var*. italic microgreens were able to have a hypoglycemic effect and enhanced lipid profile in type 2 diabetic mice (Ma et al. 2022). Furthermore, *Raphanus sativus* microgreens reduced serum sugar levels in rats with diabetes (Mohamed et al. 2023). The in vitro assays using fenugreek microgreens showed antidiabetic potentials by inhibiting α‐amylase and enhancing glucose uptake in cell lines (Wadhawan, Tripathi, and Gautam 2018). In our previous study, we identified phytochemicals with antidiabetic and hypocholesterolemic properties including α‐linoleic acid, phytol, and ethyl linolenate in the ethanolic extract of *B. carinata* microgreens (Nakakaawa et al. 2023). We further established the in vivo oral acute and subacute safety of the extract in healthy Wistar rats (Nakakaawa et al. 2023). To further investigate the potential of *B. carinata* microgreens as a nutraceutical, the aim of this study was to assess the antidiabetic effects of *Brassica carinata* microgreens ethanolic extract (BMEE) in type 2 diabetic Wistar rats.

## 2 Materials and Methods

### 2.1 Preparation of Plant Material and Extract

*B. carinata* microgreens were grown using a capillary wick irrigation system under controlled conditions in a greenhouse at Jomo Kenyatta University of Agriculture and Technology (JKUAT), Kenya (1.0912°S, 37.0117°E). Fourteen days post‐sowing, microgreens were harvested and identification was done by a taxonomist at JKUAT and a voucher specimen (JMW/JKUAT/BOT/H001) was preserved in the JKUAT herbarium. Samples underwent freeze‐drying at −46°C for 24 h, followed by pulverization into fine powder and storage in a sealed Ziplock bags until use. Two hundred and forty grams (240 g) of fresh microgreens was harvested, and sixty grams (60 g) of powder was obtained giving a ratio of 1:4 between the fresh sample and the powdered sample. *B. carinata* microgreens ethanol extract (BMEE) was obtained by cold maceration as described by (Nakakaawa et al. 2023). Sixty grams (60 g) of finely ground *B. carinata* microgreen powder was immersed in 70% ethanol at a ratio of 1:6. The mixture was then agitated on a shaker for 72 h at room temperature and filtered using Whatman filter paper no. 1.

### 2.2 Experimental Animals

Healthy 8‐ to 12‐week‐old male Wistar rats weighing between 150 and 250 g were purchased from Small Animal Facility for Research and Innovation (SAFARI), JKUAT. Animals were kept in standard well‐ventilated cages and kept at 12 h light/dark cycle at 20°C–23°C with access to commercial food pellets and water ad *libitum*.

### 2.3 Experimental Design

After a 1‐week acclimatization period, 50 rats with normal blood glucose levels (4–8 mmol/L) were used for the normoglycemic and oral glucose‐loaded assays, 25 rats per assay. The animals were fasted overnight for 12 h and then randomly divided into five groups (five rats per group). The negative control group received distilled water, while the test groups received different dosages of BMEE as follows: 100, 250, or 500 mg/kg body weight, respectively, optimized from our previous study (Nakakaawa et al. 2023). The positive control group was treated orally with 5 mg/kg glibenclamide (GLC) (Birru, Abdelwuhab, and Shewamene 2015).

Diabetes was induced by administering a high‐fat diet along with a low dose of streptozotocin according to (Chege et al. 2019) with modification. Animals under the experiment were fed on a high‐fat and high‐fructose diet (HF/HF) ad libitum for 8 weeks. The diet rich in fat was prepared by adding 40 g of lard–100 g of standard chow pellets (Unga Feeds Limited, Nairobi). The high‐fructose diet contained 20% w/v fructose solution.

After 8 weeks of HF/HF diet, a low dose of streptozotocin (40 mg/kg) (Glentham Life Sciences Ltd., UK) dissolved in 0.1 M citrate buffer (pH 4.5) intraperitoneally administered to rats that were fasted for 8 h from 7:00 a.m. to 3:00 p.m. Fasting blood glucose (FBG) levels were measured 10 days after STZ injection using an instant glucometer and strips (Accu‐chek Instant Roche Diabetes Care, South Africa). Animals with FBG levels exceeding 16 mmol/L were considered diabetic, included in the study and were maintained on a high‐fat diet. Animals in the normal control group received injections with 0.1 M citrate buffer (pH 4.5) and were maintained on standard food throughout the experiment.

Following the successful induction of T2DM, rats were randomly distributed into five groups with five animals each. Normal control (NC) received distilled water, diabetic control (DC) received distilled water, and diabetic groups were treated with 250, 500 mg/kg BMEE and glibenclamide 20 mg/kg. All the respective treatments were administered orally via gavage at 1:00 p.m. daily for 28 days. On the 29th day, upon completion of the experiment, all the animals underwent 8 h fasting period before being euthanized under anesthesia using CO_2_. Blood samples were then obtained via cardiac puncture using a syringe and needle, and collected into anticoagulant‐free tubes for serum lipid profile and serum insulin analysis. The livers were isolated from the rats for further analysis to measure gene expression.

### 2.4 Effect of BMEE on Blood Glucose Levels (BGL) in Normoglycemic and Oral Glucose‐Loaded Rats

For the normoglycemic assay, blood samples were collected to determine baseline BGL using a glucometer (Accu‐chek Instant Roche Diabetes Care, South Africa Ltd) at 0 h (before treatment), and then at 1, 2, 3, and 4 h post‐treatment. For the oral glucose‐loaded assay, dosages of BMEE or GLC were orally administered to the animals. After 30 mins of administration, 2 g/kg of glucose solution was administered orally to each rat. Blood sugar levels were determined at 0 min as a baseline, and then at 30, 60, 120, and 180 mins after glucose administration using a glucometer (Kifle 2020).

### 2.5 Measurement of Fasting Blood Glucose Levels (FBG) and Oral Glucose Tolerance Test (OGTT) in Diabetic Rats

All the animals were subjected to a morning fast for 6 h from 7:00 a.m. to 1:00 p.m., and fasting blood glucose levels were determined on a weekly basis from the tail vein throughout the entire study (Alene et al. 2020). Glucose tolerance tests were performed orally according to (He et al. 2020). The rats underwent a 6‐h fasting period before the test. Following the oral administration of the extract for 30 min, each rat received an oral dose of 2 g/kg of glucose solution. Blood sugar levels were determined at 0 min as a baseline, and then at 30, 60, 120, and 180 mins after administration of glucose.

#### 2.5.1 Serum Insulin Levels and Lipid Profile Determination

Sera were obtained by allowing the blood withdrawn from the animals to coagulate for 1 h followed by centrifugation at 5000g for 10 min. The enzyme‐linked immunosorbent assay (ELISA) method using a rat insulin kit (Solarbio Science & Technology Co. Ltd., Beijing, China) was used to determine serum insulin levels according to the manufacturer's protocol. The calculation of the Homeostasis Model Assessment of Insulin Resistance (HOMA‐IR) was performed using the following formula: HOMA‐IR = (fasting insulin in mU/L) multiplied by (fasting glucose in mmol/L) and divided by 22.5 (Abid et al. 2021). The levels of serum lipid parameters such as triglyceride (TG), total cholesterol (TC), low‐density lipoprotein (LDL), and high‐density lipoprotein (HDL) were assessed utilizing the Lisa 300 plus Chemistry analyzer (Biocode Hycel; Bio‐techne, Minneapolis, USA).

### 2.6 Messenger RNA Expression Levels of *IRS‐1*, *GLUT‐2*, and *NFKβ* in the Liver Tissues

Trizol method was used to extract total RNA from liver tissues followed by purification utilizing the Directzol RNA mini prep method (Zymo Research, USA) following the manufacturer's protocol. ProtoScript first strand cDNA synthesis kit (New England Biolabs Inc.) was utilized to synthesize complementary DNA (cDNA). Quantitative Real‐Time Polymerase Chain Reaction (qPCR) was done in triplicates using Luna universal qPCR master mix (New England Biolabs Inc.) on qTOWER3 84 (Analytik Jena, Germany). Primer sequences are listed in Table 1. All primers were synthesized by Macrogen Europe B.V. (the Netherlands). The fold change in gene expression was calculated using 2^−ΔΔ*C*^_t_ method.

**TABLE 1:** Primers for PCR amplification.

Target gene	Primer 5′–3′ sequence	NCBI accession
IRS‐1	F‐CGTCACAGGCAGAATGAAAGACC	NM_012969.2
	R‐ACGTGAGGTCCTGGTTGTGAAT	
GLUT‐2	F‐TAGTCAGATTGCTGGCCTCAGCTT	NM-012879.2
	R‐TTGCCCTGACTTCCTCTTCCAACT	
NFKβ	F‐ATCTTCAACATGGCAGACGA	NM-001276711.1
	R‐GCGGAATCGAAATCCTCTCT	
GAPDH	F‐AAGATGGTGAAGGTCGGTGT	AF-106860.2
	R‐TGACTGTGCCGTTGAACTTG	

### 2.7 Statistical Analyses

All data were presented as mean ± standard error of the mean (SEM). The difference between treated and control groups was determined by one‐way analysis of variance (ANOVA) followed by Tukey's test. Values were considered significant at *p* < 0.05. All statistical analysis and preparation of graphs were done using GraphPad Prism software version 8.4.3. for Windows. The area under the curve (AUC) for the oral glucose tolerance tests was calculated using GraphPad Prism software version 8.4.3. for Windows.

## 3 Results

### 3.1 BMEE Did Not Affect Blood Glucose Levels (BGLs) of Normoglycemic Rats

A single administration of BMEE caused no significant alteration in blood glucose levels in contrast to the untreated group at all‐time points (*p* > 0.05) (Table 2). The glibenclamide‐treated group showed a significant BGL reduction (*p* < 0.05), in contrast to the control group. Within‐group analysis showed that the glibenclamide‐treated group significantly reduced the BGLs when compared to all the BMEE dose levels at all‐time points (*p* < 0.0001).

**TABLE 2:** Effect of BMEE on blood glucose level of normoglycemic rats.

Blood glucose levels, mmol/L				
Group	0 h	1 h	2 h	4 h
Control	5 ± 0.23	5.48 ± 0.16	5.46 ± 0.4	5.76 ± 0.31
GLC5	4.96 ± 0.11	2.88 ± 0.33^a,b,c,d^	2.5 ± 0.18^a,b,c,d^	2.76 ± 0.42^a,b,c,d^
BMEE 100	4.98 ± 0.17	4.92 ± 0.15	4.92 ± 0.53	4.8 ± 0.21
BMEE 250	4.82 ± 0.18	4.94 ± 0.31	4.82 ± 0.22	4.72 ± 0.18
BMEE 500	4.98 ± 0.19	5 ± 0.23	4.92 ± 0.51	4.8 ± 0.44

*Note:* Values are expressed as mean ± SEM (standard error mean); error bars represent SEM (*n* = 5 rats/group). Significant difference when compared to (a) negative control, (b) BMEE 100 mg/kg, (c) BMEE 250 mg/kg and (d) BMEE 500 mg/kg.

### 3.2 Effect of BMEE on Blood Glucose Level in Oral Glucose‐Loaded Rats

The effect of BMEE on oral glucose tolerance test (OGTT) in oral glucose‐loaded rats is shown in Table 3. After 180 min of glucose loading, all animals pretreated with BMEE or GLC showed reduced BGLs to a level comparable to the baseline. BMEE 500 mg/kg treated and the glibenclamide‐treated groups showed significant reductions in BGLs when compared to the untreated group (*p* = 0.039 and *p* = 0.002, respectively). Analysis showed that GLC significantly reduced BGLs within the first 30 min of glucose loading compared to BMEE 100 mg/kg (*p* = 0.043) and BMEE 250 mg/kg (*p* = 0.036).

**TABLE 3:** Effect of BMEE on blood glucose level in oral glucose‐loaded rats.

Blood glucose levels, mmol/L				
Group	0 min	30 min	60 min	180 min
Control	5.24 ± 0.46	7.86 ± 0.6	6.8 ± 0.62	5.48 ± 0.29
GLC5	5.5 ± 0.13	4.78 ± 0.56^a,b,c^	4.56 ± 0.67^a,c^	3.56 ± 0.35^a,b^
BMEE 100	5.5 ± 0.22	7.08 ± 0.34	6.12 ± 0.19	5.3 ± 0.27
BMEE 250	5 ± 0.07	7.14 ± 0.74	6.86 ± 0.26	4.8 ± 0.3
BMEE 500	5.28 ± 0.33	6.02 ± 0.27	5.56 ± 0.58	4.1 ± 0.35^a^

*Note:* Values are expressed as mean ± SEM (standard error mean); error bars represent SEM (*n* = 5 rats/group). Significant difference when compared to (a) negative control group, (b) BMEE 100 mg/kg, (c) BMEE 250 mg/kg.

### 3.3 BMEE Reduced Fasting Blood Glucose Levels (FBGLs) in Diabetic Rats

Throughout the 4 weeks of treatment, the diabetic group (DC), glibenclamide‐treated group (GLC5), BMEE 250 and 500 mg/kg treatment groups showed significantly high FBGLs in comparison with the NC group (*p* < 0.0001) (Table 4). At 14 days of treatment, BMEE treatments did not show any significant difference in FBGLs in comparison with the diabetic control group (*p* = 0.357 and *p* = 0.126) while GLC5 lowered FBGLs significantly (*p* = 0.005). However, subsequent assessments on Days 21 and 28 showed a significant reduction in FBGLs in groups treated with BMEE 250 mg/kg, BMEE 500 mg/kg and GLC5 (*p* < 0.0001) when compared to the diabetic group. No significant difference between the GLC5 and BMEE‐treated groups at these time points except on Day 28 where GLC5 showed a significant reduction in FBGLs than BMEE 250 mg/kg (*p* = 0.004).

**TABLE 4:** Effect of BMEE on fasting blood glucose levels in diabetic rats.

Blood glucose levels, mmol/L					
Group	0 day	7 days	14 days	21 days	28 days
NC	4.98 ± 0.17	4.92 ± 0.15	4.92 ± 0.53	4.8 ± 0.21	4.32 ± 0.14
DC	25.96 ± 0.52	22.22 ± 0.45^a^	22.52 ± 0.59^a^	23.98 ± 0.79^a^	22.12 ± 0.27^a^
GLC5	22.26 ± 0.31	18.5 ± 0.89^a^	18.3 ± 1.02^a,b^	17.84 ± 0.37^a,b^	15.64 ± 1.19^a,b,c^
BMEE 250	25.6 ± 1.21	20.42 ± 0.49^a^	20.56 ± 0.97^a^	19.28 ± 0.73^a,b^	18.12 ± 0.21^a,b^
BMEE 500	22.96 ± 1.73	19.68 ± 1.61^a^	19.9 ± 0.26^a^	18.1 ± 0.36^a,b^	17.04 ± 0.47^a,b^

*Note:* Values are expressed as mean ± SEM (standard error mean); error bars represent SEM (*n* = 5 rats/group). Significant difference when compared to (a) NC group, (b) the diabetic group, (c) BMEE 250 mg/kg group.

### 3.4 BMEE Improved Oral Glucose Tolerance Tests in Diabetic Rats

**FIGURE 1:**
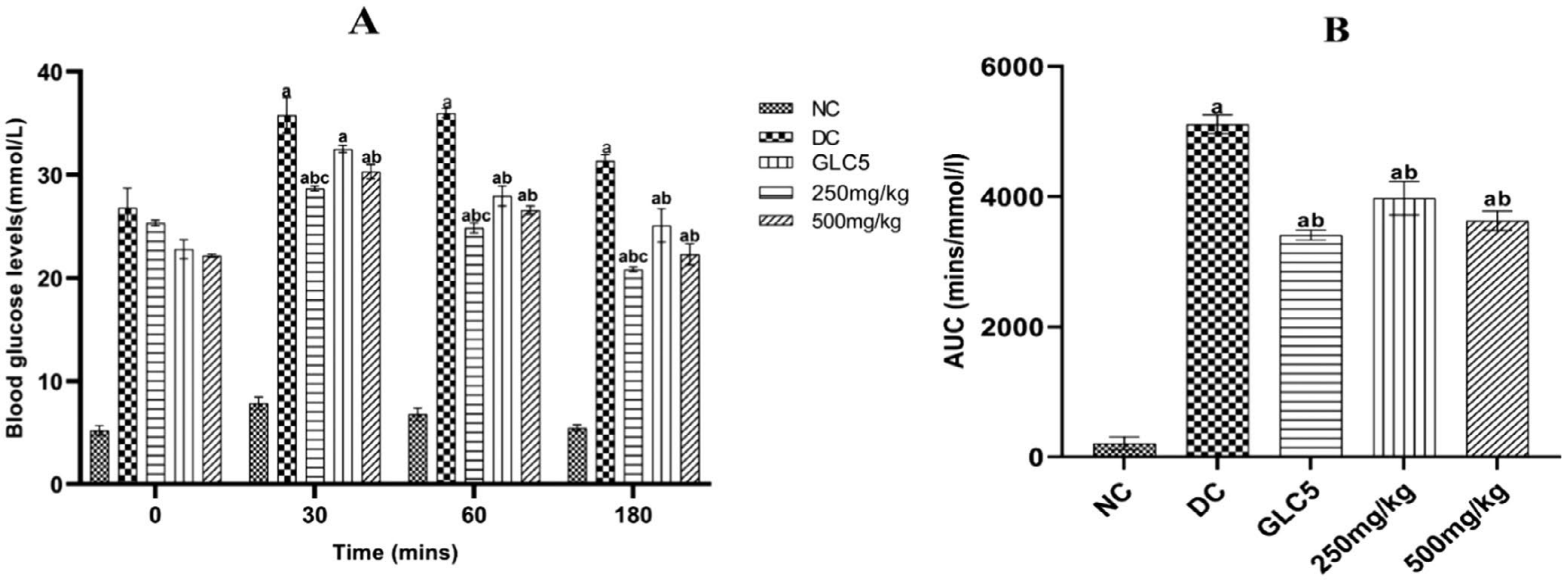
Effect of BMEE on oral glucose tolerance tests (OGTT) and AUC of OGTT in diabetic rats. (A) Effect of BMEE on oral glucose‐loaded diabetic rats. (B) AUC of oral glucose tolerance in diabetic rats. Values are expressed as mean ± SEM (standard error mean); error bars represent SEM (*n* = 5 rats/group). Significant difference when compared to (a) normal control group, (b) the diabetic group, (c) BMEE 250 mg/kg group.

BMEE effect on oral glucose tolerance test and area under the curve (AUC) is shown in Figure 1. Administration of 2 g/kg of glucose solution orally to diabetic rats 30 min administration of distilled water, test extracts, and glibenclamide increased blood glucose levels. Thirty minutes after glucose loading, BMEE 500 mg/kg and GLC5 significantly lowered BGLs in comparison with the untreated diabetic group (*p* = 0.002 and *p* = 0.0001, respectively). However, after 60 and 180 min, both dosages of BMEE and GLC5 significantly reduced BGLs when compared to the diabetic group (*p* < 0.0001 and *p* = 0.0005).

### 3.5 BMEE Improved Insulin Sensitization and Reduces Insulin Resistance in Diabetic Rats

**FIGURE 2:**
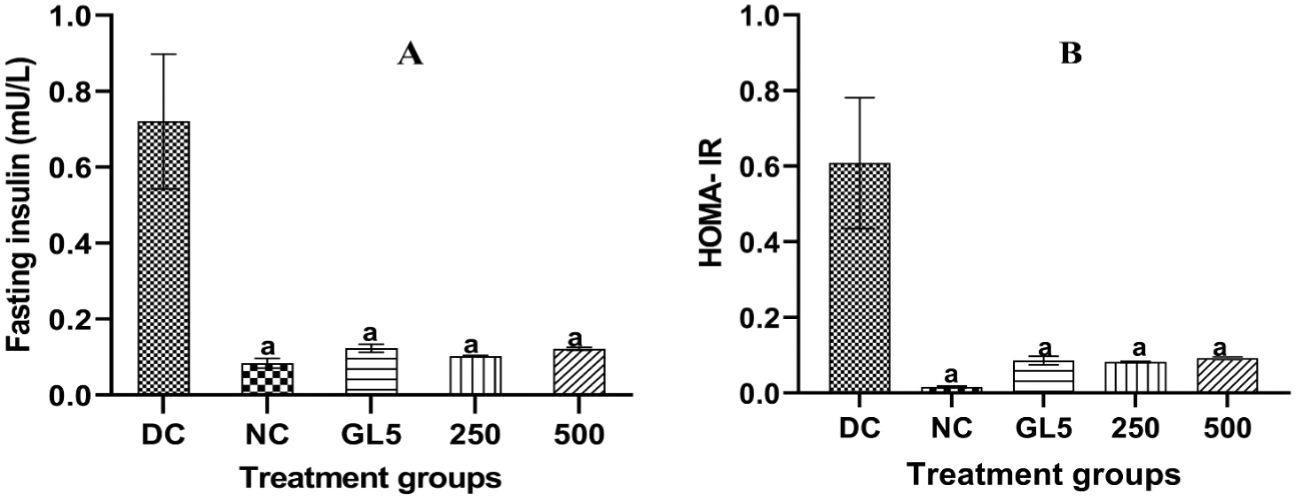
Effect of BMEE on fasting insulin levels and HOMA‐IR. (A) Fasting serum insulin levels of diabetic rats. (B) HOMA‐IR of diabetic rats. Values are expressed as mean ± SEM (standard error mean); error bars represent SEM (*n* = 5 rats/group). (a) Significant difference when compared to the DC group.

BMEE effect on fasting serum insulin levels and the homeostasis model of assessment of Insulin Resistance (HOMA‐IR) is presented in Figure 2. Animals in the diabetic control (DC) showed severe insulin resistance manifested by elevations in the fasting serum insulin levels. However, a significant reduction in the insulin levels was observed when the normal control (*p* = 0.0001), glibenclamide (*p* = 0.0003), BMEE 250 mg/kg (*p* = 0.0002), and BMEE 500 mg/kg (*p* = 0.0003) groups in comparison with the diabetic control. A significant reduction in the HOMA‐IR levels was also observed when the normal control (*p* = 0.0002), GLC (*p* = 0.001), BMEE 250 mg/kg (*p* = 0.0009), and BMEE 500 mg/kg (*p* = 0.0012) groups were contrasted with the diabetic control.

### 3.6 BMEE Improved Lipid Metabolism

**FIGURE 3:**
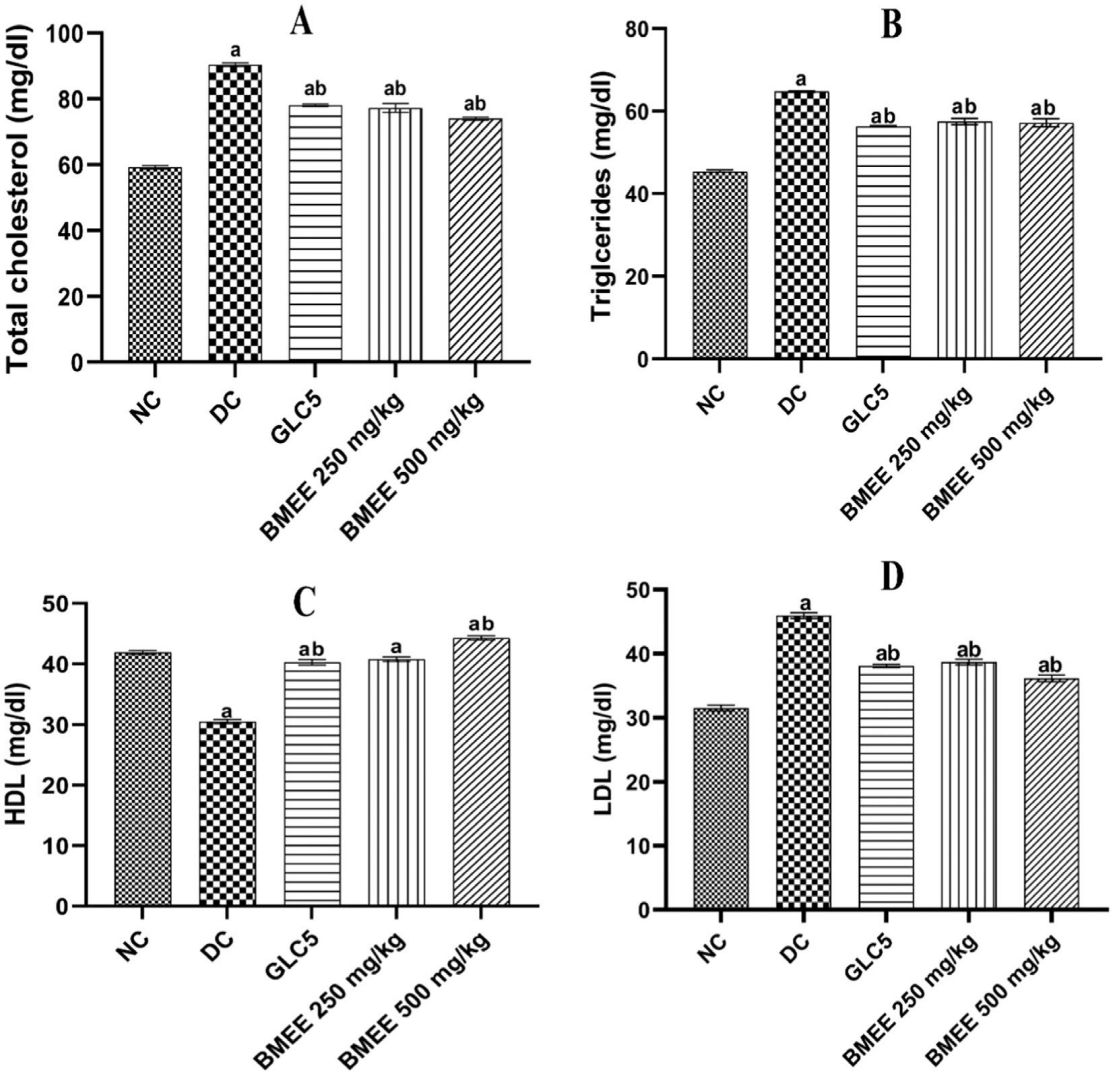
Effect of BMEE on serum lipid profile of diabetic rats. (A) TC levels of diabetic rats. (B) TG levels of diabetic rats. (C) HDL levels of diabetic rats. (D) LDL levels of diabetic rats. Values are expressed as mean ± SEM (standard error mean); error bars represent SEM (*n* = 5 rats/group). Significant difference when compared to (a) NC group, (b) the diabetic group.

BMEE effect on serum lipid profile is shown in Figure 3. Between groups analysis revealed a significant reduction in the total cholesterol (TC) when GLC5, BMEE 250 and 500 mg/kg were compared to the diabetic group (*p* < 0.0001). Treatment with GLC5, BMEE 250 and 500 mg/kg was able to significantly lower the triglycerides (TG) levels in comparison with the diabetic group (*p* < 0.0001). Analysis for high‐density lipoprotein (HDL) showed significantly increased levels in the GLC5, BMEE 250 and BMEE 500 mg/kg groups when compared to the diabetic group (*p* < 0.001). When LDL levels of GLC5, BMEE 250 and 500 mg/kg were compared to those of the diabetic group, a significant decrease was observed (*p* < 0.0001).

### 3.7 BMEE Treatment Increased *IRS* ‐*1* and *GLUT* ‐*2* Expression While Reducing *NFKβ* Expression

**FIGURE 4:**
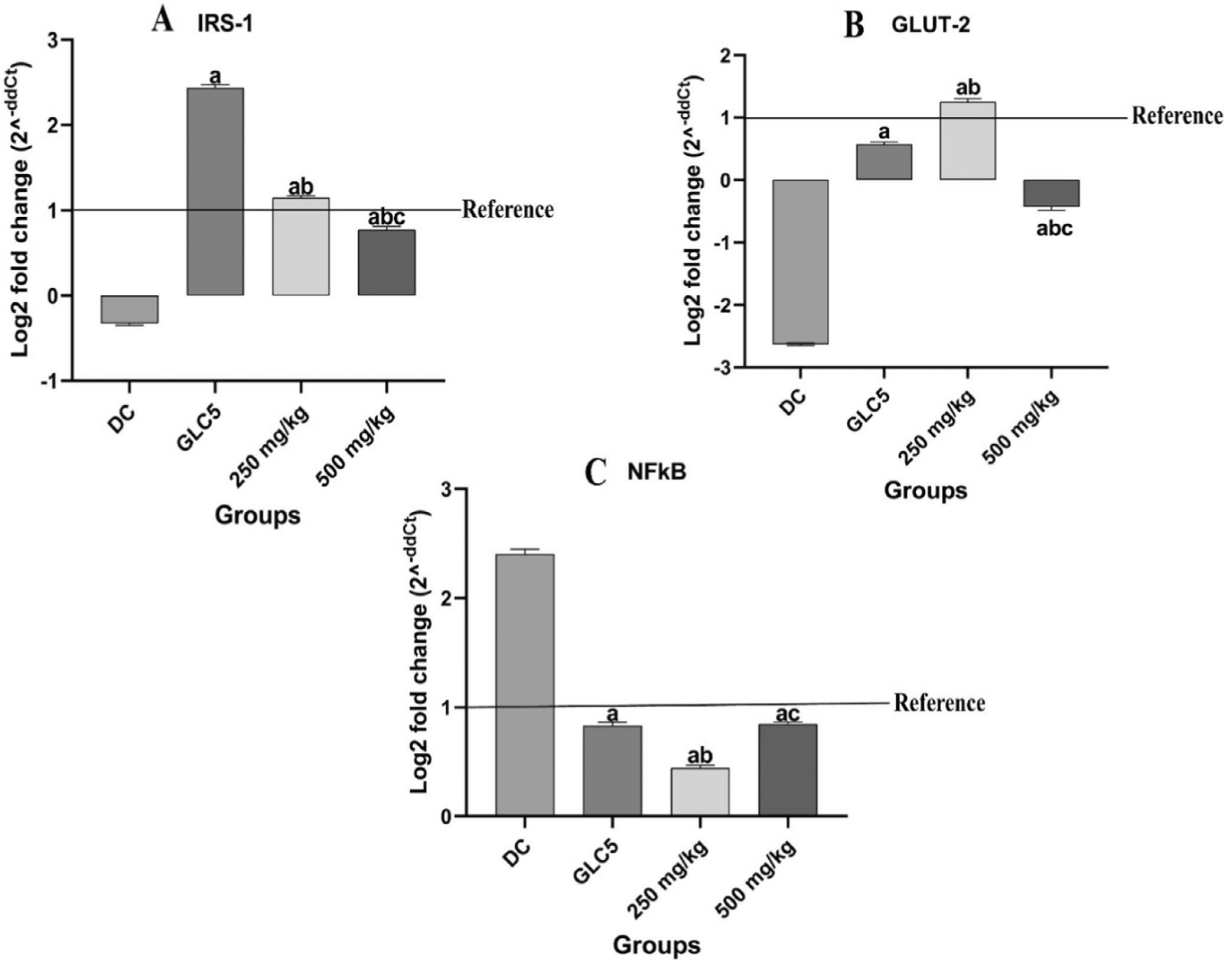
Effect of BMEE on relative gene expression of *IRS‐1*, *GLUT‐2* and *NFKB* in the liver. (A) *IRS‐1* gene expression. (B) *GLUT‐2* gene expression. (C) *NFKβ* gene expression. Values are expressed as mean ± SEM (standard error mean): error bars represent SEM (*n* = 5 rats/group). Significant difference when compared to (a) DC, (b) GLC5 group, (c) 250 mg/kg group. Reference is the value at which there is no change in gene expression (log_2_).

The effect of BMEE on the gene expression of Insulin receptor substrate 1, Glucose transporter 2, and Nuclear Factor Kappa Light Chain Enhancer of Activated B cells (*IRS‐1*, *GLUT‐2*, and *NFKβ*) genes is shown in Figure 4. The *IRS*‐*1* gene was significantly downregulated (fold change = −0.32) in the diabetic group compared to the treatment groups (*p* < 0.0001). Within the treatment groups, a significant upregulation (*p* < 0.0001) of *IRS*‐*1* was observed in animals treated with GLC and 250 mg/kg BMEE with a relative fold change of 2.43 and 1.14, respectively, when compared to the diabetic group. Notably, BMEE did not show a linear dosage‐dependent effect on the regulation of *IRS*‐*1* as a downregulation (fold change = 0.77) was observed in animals treated with 500 mg/kg BMEE compared to 250 mg/kg (*p* = 0.01). *GLUT*‐*2* gene was significantly downregulated in the diabetic group with a relative fold change of −2.63. In the treatment groups, GLC and 500 mg/kg BMEE treatments caused a downregulation of the gene with a fold change of 0.56 and −0.42, respectively. However, the expression of the *GLUT*‐*2* gene in GLC‐treated animals was significantly higher (*p* = 0.008) than in the 500 mg/kg BMEE group. On the other hand, animals treated with 250 mg/kg BMEE showed a relatively similar gene expression level (fold change = 1.25) to the healthy control group. Similar to *IRS*‐*1*, a *GLUT*‐*2* expression was not dosage dependent in animals treated with BMEE. Despite the differences in the treatment groups, *GLUT‐2* expression in all treated animals varied significantly from the diabetic group (*p* < 0.0001).

The pro‐inflammatory gene, *NFKβ*, was upregulated in the diabetic group with a fold change of 2.40, but downregulated in all treatment groups with fold changes of 0.82, 0.44, and 0.84 in the GLC5, 250 and 500 mg/kg groups, respectively. Statistical analysis showed that treatment with GLC5, 500, and 250 mg/kg significantly downregulated *NFKβ* expression when compared to the diabetic group (*p* < 0.0001).

## 4 Discussion

Type 2 diabetes is a chronic metabolic disorder with long‐term detrimental impact on human health, and the current pharmacological interventions pose severe side effects hence fueling efforts in search of effective and safe alternative therapeutic interventions (Khan et al. 2020). Microgreens, for instance, are abundant in minerals, nutrients, and antioxidants and have recently gained popularity in the prevention of chronic diseases (Mohamed et al. 2022). The current study evaluated the antidiabetic potentials of *B. carinata* microgreens ethanol extract (BMEE) in type 2 diabetic rats.

The effects of BMEE on blood glucose levels (BGLs) in normoglycemic and oral glucose‐loaded nondiabetic rats were first assessed. The effect of a substance on BGLs in normoglycemic animals is used to assess its hypoglycemic activities (Boye et al. 2020). From our study, we show that BMEE does not exert a hypoglycemic effect on normoglycemic rats unlike glibenclamide, which caused a decline in BGLs within 1 h of administration. While this study did not definitively investigate the mechanism behind the unchanged BGLs in normoglycemic animals by BMEE, it could be attributed to the sustenance of glucose metabolism within the range of normal homeostasis. This strongly suggests that BMEE does not cause hypoglycemia in animals with healthy and intact pancreas, possibly due to undisrupted normal pancreatic function in the animals. Therefore, the sustenance of normal glucose homeostasis by BMEE is quite beneficiary to its potential use as a nutraceutical (Birru, Abdelwuhab, and Shewamene 2015).

The oral glucose tolerance test (OGTT) is a commonly employed diagnostic tool for impaired glucose tolerance (IGT). In animal studies, the OGTT is utilized to evaluate the extent of diabetes and to examine the impact of drugs effects on glucose metabolism (Ye et al. 2012). The area under the curve (AUC) is computed from the OGTT data and used to quantify the total increase in blood glucose (Kuo et al. 2021). Consequently, the reduction in AUC indicates the improvement in IGT in animals. Oral glucose loading causes physiological hyperglycemia, and this was confirmed in this study by the observed rise in BGLs of the animals 30 min after glucose loading in both normoglycemic and diabetic animals. Contrary to the normoglycemic rats, BMEE lowered BGLs in glucose‐loaded nondiabetic rats in a time‐dependent manner, and significantly after 60 min of administration in animals that received 500 mg/kg. The contrasting action of BMEE in glucose‐loaded rats may be associated with previous reports that impairment of glucose tolerance in hyperglycemic rats may cause a stronger suppression of glucose absorption compared to normoglycemic animals. Although there was a delayed onset of action of BMEE compared to glibenclamide, there was no significant difference in the extent of decline in glycemic peaks exerted by both treatments.

Furthermore, we demonstrated that BMEE at a dosage of 500 mg/kg decreased BGLs in glucose‐loaded diabetic animals within 30 min after loading, an earlier onset compared to glucose‐loaded nondiabetic animals. This further supports our speculation that BMEE may selectively lower BGLs in hyperglycemic conditions and in correlation with increasing impairment of glucose tolerance. Our results also show the potential of a lower dosage (250 mg/kg) of BMEE to lower glycemic peaks within 60 min post glucose loading. Noteworthy, there was no significant difference between the actions of glibenclamide and BMEE 500 mg/kg. The AUC of BMEE pretreated animals at both dosages was significantly lower than that of the diabetic group suggesting that BMEE can alleviate IGT, probably due to enhanced glucose utilization and improved glycemic control (Cheng 2018).

Since 250 and 500 mg/kg dosages of BMEE showed notable anti‐hyperglycemic activities in the OGTT assays, the doses were employed in a continuous 28‐day assessment of fasting blood glucose levels (FBGLs) using streptozotocin‐induced T2DM rats. Continuous administration of BMEE dosages produced a statistically significant dose‐dependent antidiabetic effect after 14 days of treatment, a delayed onset when compared to glibenclamide's actions observed after 7 days. However, there was no significant difference between the effects of BMEE 500 mg/kg and glibenclamide at the end of the treatment course. These provide evidence that BMEE not only lowered postprandial BGLs, but also FBGLs in diabetic rats during the period tested. Increased FBGLs also known as hyperglycemia is an important characteristic feature in diabetes mellitus (Zhou et al. 2023) and plants that lower FBGLs attract great interest in the management of T2DM (Alene et al. 2020). The significant anti‐hyperglycemic effect of BMEE could be attributed to the presence of antidiabetic compounds phytocompounds like Phytol and (3β)‐Stigmast‐8(14)‐en‐3.‐ol (Matsuda et al. 2017; Sujatha et al. 2010) that were identified from our previous study (Nakakaawa et al. 2023). The anti‐hyperglycemic activity of BMEE is also supported by previous studies on plants belonging to the *Brassicaceae* family like *Brassica nigra* , *Brassica napus*, and *Brassica juncea* that showed antidiabetic activities in rats, mice, and humans (Chio et al. 2018; Tiwari and Kumar 2018; Yun et al. 2019).

Insulin resistance, the hallmark of diabetes and obesity is a crucial component in the pathogenesis of type 2 diabetes with a significant role in the onset of long‐term complications like hypertension and hyperlipidemia as well as the development of hyperglycemia in non‐insulin‐dependent diabetes (Chege et al. 2019). The Homeostasis model of assessment of insulin resistance (HOMA‐IR) index has been used to assess both beta‐cell function and insulin resistance in pre‐clinical investigations of people with both glucose intolerance, mild to severe diabetes, and other insulin‐resistant diseases (Antunes et al. 2016). BMEE demonstrated strong anti‐hyperinsulinemia by lowering the insulin levels. These effects could be due to increased insulin sensitivity and a decrease in insulin resistance through a significant impact on serum insulin levels as well on the HOMA‐IR levels. We speculate that the observed anti‐hyperglycemic effects of BMEE could be secondary to its reduction in insulin resistance.

A high‐fat diet causes abnormalities in lipid metabolism and profile like higher levels of triglyceride (TG), total cholesterol (TC), low‐density lipoprotein (LDL), and lower levels of high‐density lipoprotein (HDL), in type 2 diabetic rats. This could be due to increased mobilization of free fatty acids from peripheral deposits to central blood circulation (Khattab et al. 2022; Mohamed et al. 2022). BMEE at 250 and 500 mg/kg possessed a significant antidyslipidemic effects by decreasing TC, LDL, TG, and increasing HDL‐cholesterol. Stigmast‐8(14)‐en‐3.beta.‐ol, Ethyl 9,12,15‐octadecatrienoate and 9,12,15‐octadecatrienoic acid are some of the phytocompounds identified in BMEE (Nakakaawa et al. 2023), and they have been reported to improve the lipid metabolism and possess antihyperlipidemic properties (Guerrero, Vargas, and Petricevich 2017; Pinto et al. 2017). Results from this study are like the report of Huang et al. (2016) that reported microgreens of *Brassica oleracea* L. var. capitata at a dosage level of 200 and 400 mg/kg lowered lipid and cholesterol levels in mice fed on a high‐fat diet.

To investigate the molecular mechanism of action of BMEE, we evaluated the gene expression of important gene markers in glucose/insulin metabolism, glucose transporter 2 (*GLUT‐2*) and insulin receptor substrate 1 (*IRS‐1*), and pro‐inflammatory markers indicative of the persistence of hyperglycemic conditions, *NFKβ*, in the liver. In diabetes, reactive oxygen species (ROS) production is increased due to hyperglycemia and increased free fatty acids. According to studies, ROS can interfere with the insulin signaling pathway by affecting *IRS* and *GLUT* functions, thus, lowering glucose uptake (He et al. 2020). Also, the liver is crucial in glucose and lipid homeostasis via hepatic insulin signaling (Honma et al. 2018) and is usually affected by long‐term insulin resistance and hyperglycemia situations (Barata et al. 2019). Hepatic insulin resistance, a hallmark pathogenic feature of T2DM, is caused by decreased hepatocellular insulin receptor substrate. In normal physiology, the binding of insulin to the insulin receptor causes its phosphorylation hence initiating insulin action (Chen et al. 2022). During diabetes type 2, there is an inactivation and downregulation of *IRS*‐*1* which was confirmed from our study where the gene expression of *IRS‐1* was significantly downregulated in the diabetic group but significantly upregulated with BMEE and glibenclamide treatment. This suggests that BMEE reduces hepatic insulin resistance by upregulating *IRS‐1* and increasing insulin action.

The expression of the *GLUT‐2* gene is downregulated in diabetic conditions; this was also observed in the diabetic group from our study. Treatment with BMEE at 250 mg/kg and glibenclamide, however, significantly increased *GLUT‐2* expression, suggesting increase in hepatic glucose uptake resulting in reduced FBGLs. *GLUT‐2* is the major glucose transporter in hepatocytes of rodents and humans and controls the first step of glucose metabolism in the liver (Thorens 2015). Its expression is crucial for glucose sensing and homeostasis; therefore, its inactivation in the liver could impair glucose‐stimulated insulin secretion (Chen et al. 2022). From our study, persistent hyperglycemia led to the upregulation of *NFKβ* in diabetic control animals while BMEE treatment was able to down regulate the expression of *NFKβ* significantly indicating improved glucose tolerance and reduced insulin resistance in the treated diabetic animals. In the early phases of diabetes type 2, *NFKβ* signaling and the liver's synthesis of pro‐inflammatory mediators lead to insulin resistance and pathogenesis of diabetic vascular complications (Meyerovich, Ortis, and Cardozo 2018).

An interesting reverse dose dependence was observed in the gene expression patterns of *IRS‐1*, *GLUT‐2* and *NFKβ* in response to BMEE treatment at 250 and 500 mg/kg. The lower dosage of 250 mg/kg exerted stronger antidiabetic effects through regulation of mRNA expression when compared to the higher dosage. While further pharmacokinetic studies will extensively investigate this, we speculate that this could be associated with the rate of drug distribution and absorption. Pharmacokinetic studies of drug candidates sometimes reveal delayed distribution of the drug to the site of action as the concentration or dose increase, thus causing delayed and attenuated actions (Currie 2018).

## 5 Conclusion

The research findings provided compelling evidence that BMEE has promising therapeutic potential in the management of diabetes type 2. The extract exhibited noteworthy capacity to improve various critical health indicators in diabetic rats. Notably, BMEE showed antidiabetic activities without affecting normal blood glucose levels. BMEE can reduce hepatic insulin resistance and the same time improve hepatic glucose uptake via the insulin and the NF‐kB signaling pathways by increasing IRS‐1 and GLUT‐2 gene expression while reducing gene expression of NFkB in the liver. The lipidomic analysis to predict the possible connections between potential BMEE components and T2DM was not done and the effect of BMEE on other biochemical markers like glycated hemoglobin (HbA1c), C‐peptide, ketone bodies, and liver enzymes like ALT and AST needs to be done.

## Author Contributions

**Lilian Nakakaawa:** conceptualization (lead), data curation (lead), funding acquisition (lead), methodology (equal), validation (equal), visualization (equal), writing – original draft (lead), writing – review and editing (equal). **Ifeoluwa D. Gbala:** conceptualization (equal), data curation (equal), methodology (equal), supervision (equal), validation (equal), visualization (equal), writing – review and editing (equal). **Joel L. Bargul:** conceptualization (equal), data curation (equal), methodology (equal), supervision (lead), validation (equal), visualization (equal), writing – review and editing (equal). **Xavier Cheseto:** data curation (equal), investigation (equal), methodology (equal), supervision (equal), validation (equal), visualization (equal), writing – review and editing (equal). **John M. Wesonga:** conceptualization (equal), data curation (equal), investigation (equal), resources (equal), validation (equal), visualization (equal), writing – review and editing (equal).

## Ethics Statement

Animal experiments were conducted according to the National Institutes of Health's Guide for the Care and Use of Laboratory Animals and the Animal Research: Reporting of In Vivo Experiments (ARRIVE) guidelines, approved by the Animal Use and Care Ethics Review Committee, Mount Kenya University, Kenya (Ref. MKU/ERC/2250).

## Conflicts of Interest

The authors declare no conflicts of interest.

## Data Availability

The data that support the findings of this study are available on request from the corresponding author.
